# Creating a data collection and management platform to support measurement of adolescent HIV care transition processes within low- and middle-income countries: The GRADUATE project

**DOI:** 10.1371/journal.pgph.0002705

**Published:** 2024-08-05

**Authors:** Priscilla R. Tsondai, Mary-Ann Davies, Thida Singtoroj, Nicola Maxwell, Karl-Günter Technau, Kulkanya Chokephaibulkit, Pagakrong Lumbiganon, Annette H. Sohn, on behalf of the Global fRAmework of Data collection Used for Adolescent HIV Transition Evaluation (GRADUATE) Advisory Group

**Affiliations:** 1 Centre for Infectious Disease Epidemiology and Research, School of Public Health, University of Cape Town, Cape Town, South Africa; 2 TREAT Asia/amfAR—The Foundation for AIDS Research, Bangkok, Thailand; 3 Faculty of Health Sciences, Department of Paediatrics & Child Health, Empilweni Services and Research Unit, Rahima Moosa Mother and Child Hospital, University of the Witwatersrand, Johannesburg, South Africa; 4 Faculty of Medicine Siriraj Hospital, Department of Pediatrics, Mahidol University, Bangkok, Thailand; 5 Faculty of Medicine, Department of Pediatrics, Khon Kaen University, Khon Kaen, Thailand; Baylor College of Medicine, UNITED STATES OF AMERICA

## Abstract

Few national programs and research cohorts within low- and middle-income countries (LMICs) document transition-related processes and outcomes for adolescents and young adults living with HIV (AYLH) transitioning to adulthood. Between 2017–2020, The Global fRAmework of Data collection Used for Adolescent HIV Transition Evaluation (GRADUATE) project convened a collaborative advisory group to identify key variables and definitions capturing the process, predictors, and outcomes across the transition period. In total, 114 variables identified as essential to measuring AYLH transition-related data were identified and formatted into a GRADUATE Data Exchange Standard (DES), which was added to and harmonized with the existing International epidemiology Databases to Evaluate AIDS (IeDEA) DES. In 2019, the GRADUATE DES was pilot tested at four IeDEA facilities in Malawi, South Africa, and Thailand through a cross-sectional study. Upon comparing the variables to routine medical records, available data were too limited to adequately capture transition-related processes and outcomes. However, additional data collection using GRADUATE tools was feasible and improved completeness. Of the 100 (52% female) AYLH included in the pilot study, 71% had transitioned/transferred to adult care, with 42% transitioning from an adolescent-specific model of care within an integrated family clinic to having their clinic visits scheduled on a different day of the week while 58% transferred from a pediatric facility to one offering adult HIV care. While almost all (94%) had a transition-related discussion with their healthcare providers prior to the transition, we found that 69% (95% CI 49–85%) were somewhat or very satisfied/comfortable with the post-transfer clinic and the staff. Utilization of the GRADUATE DES better characterized AYLH transitioning to adulthood across LMICs, and optimally measured transition preparation activities and outcomes. Utilization of the GRADUATE DES in other settings could facilitate comparisons and identify gaps in the care of transitioning adolescents that need to be addressed.

## Introduction

Antiretroviral therapy (ART) transformed HIV to a chronic illness. An increasing number of children and adolescents living with perinatally and non-perinatally acquired HIV are surviving beyond adolescence into adulthood and having to manage HIV for the rest of their lives [1]. There are over three million adolescents and young adults living with HIV (AYLH) aged 15 to 24 years in the world today [2]. As these AYLH grow, they face a series of steps associated with transitions in their care and are expected to become increasingly responsible for their own healthcare. The goal of transition is to continue providing uninterrupted, high-quality, and developmentally appropriate healthcare services as an individual moves from adolescence to adulthood. In high-income countries, this frequently involves transferring care from a pediatric/adolescent setting to an adult HIV clinic, often following a systematic and structured process [1,3–6]. However, in low- and middle-income countries (LMICs), transition to adulthood for children living with HIV may not always involve physical movement to a different clinic or change in provider and is often not well-defined or structured [7]. Nonetheless, as they age, youth increasingly become empowered to self-manage their disease and start attending clinic visits and collecting their medications without a caregiver. The healthcare providers also begin interacting with them as adults rather than as children/adolescents, even if they are still receiving care within the same facility. This time of a person’s life may also be a vulnerable period in general and with regard to ART adherence in particular, where sudden or unplanned changes may cause care interruptions or worsen outcomes [8,9].

While research on the transition of AYLH into adult care has progressed in specific cohorts in the US and Europe [10–13], the field remains in early stages in LMICs, where the vast majority of AYLH reside. Few national programs or research cohorts in LMICs document the transition experience or its outcomes [14,15]. Whereas high-income countries may have access to research cohorts with extensive demographic and behavioral questionnaires as well as non-routine laboratory data, cohorts within LMICs have more limited data gathered in routine care. Moreover, routine data within these settings may not yet include many of the variables needed to fully characterize this population or to adequately assess transition-related processes and outcomes. There is no standard method for tracking these outcomes across the transition period which seriously limits surveillance of these targets in adolescents. Also, central to assessing and comparing transition outcomes is knowing how to optimally measure them and implementing data collection across different settings.

The Global fRAmework of Data collection Used for Adolescent HIV Transition Evaluation (GRADUATE) project aimed to create the first broadly implementable and open access data collection and management platform that would support measurement of adolescent HIV care transition processes and outcomes. We describe the processes of creating this platform, and present results from the pilot exercise conducted to assess the developed tools.

## Methods and materials

**Fig 1:**
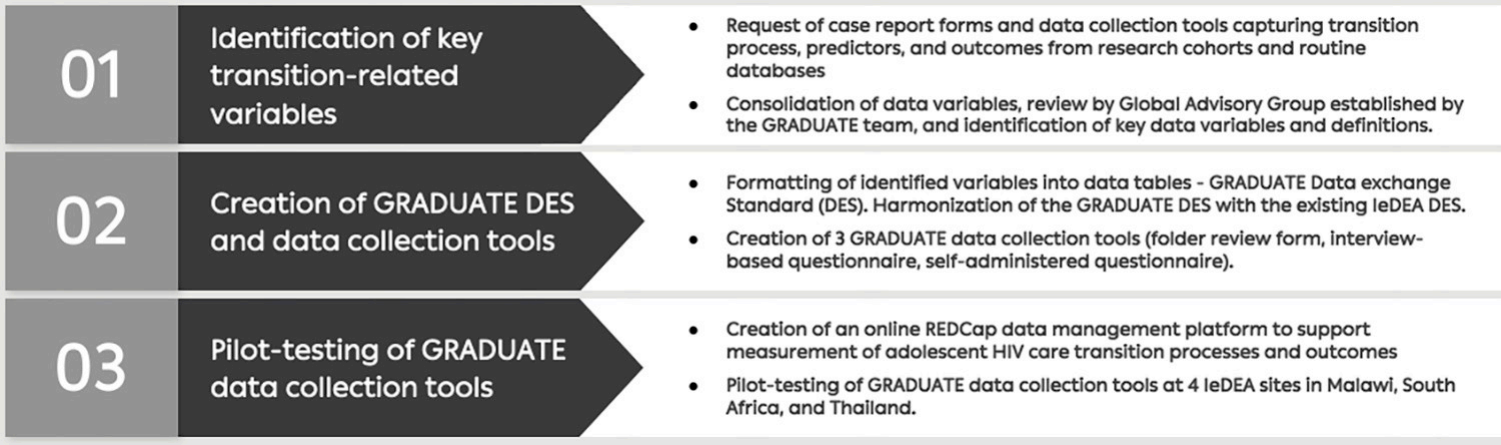
Phases of the GRADUATE project in creating a data collection and management platform to support measurement of adolescent HIV care transition processes and outcomes. CRFs–case report forms; DES–Data Exchange Standard; GRADUATE—Global fRAmework of Data collection Used for Adolescent HIV Transition Evaluation; IeDEA—International epidemiology Databases to Evaluate AIDS.

The GRADUATE project was conducted between 2017–2020 in three phases, as shown in Fig 1.

### Phase 1: Identification of key transition-related variables

Case report forms and data collection tools were requested from established research cohorts and routine databases in countries across all income settings that were actively collecting transition-related data from adolescents living with HIV, namely: the Pediatric HIV/AIDS Cohort Study (PHACS) Adolescent Master Protocol’s AMP Up cohort in the US [16], Adolescents and Adults Living with Perinatal HIV (AALPHI) cohort in the UK [17], Centre Maternel et Infantile sur le SIDA (CMIS) Mother-Child Cohort in Canada [18], Cape Town Adolescent Antiretroviral Cohort (CTAAC) in South Africa [19], and the Study of Transitioning Asian Youth (STAY) Cohort in Southeast Asia. In addition, the International epidemiology Databases to Evaluate AIDS (IeDEA) [20] Data Exchange Standard (DES) was reviewed, which is a data model for sharing observational HIV data, designed and maintained by the IeDEA Data Harmonization Working Group [21]

Variables on key demographic, socioeconomic, clinical, and laboratory aspects relating to AYLH within the received case report forms and DES were extracted. These were presented to an advisory group composed of expert clinicians, researchers, and implementers working with children and AYLH from across various country income settings, established by the GRADUATE project. The advisory group reviewed the pooled variables. Through a prioritization exercise, they evaluated each variable, rating them to distinguish between those that were essential and optional. Subsequently, they deliberated on the ratings through a consultative process, paying particular attention to those that had been deemed optional or unnecessary and that would be excluded from the list of variables. They reached consensus on the variables central to capturing characteristics of AYLH transitioning to adulthood across LMICs, as well as the processes, predictors, and outcomes across the transition period.

### Phase 2: Creation of the GRADUATE DES and data collection tools

In phase 2, selected variables were categorized into three tiers, depending on how likely they were to already be captured or how easily they could be obtained.

- Tier one or "basic core variables” were defined as those most likely to be routinely captured by HIV programs within LMICs that could be used for surveillance and program evaluations, such as those describing ART history, anthropometric measures, missed appointments, retention, transfer of care, and immunologic and virologic markers.
- Tier two or “advanced core variables” were defined as those that may not be available within routine care but should be collected whenever possible for assessing clinical outcomes within dedicated cohorts. These include variables capturing transition preparation, disclosure, orphanhood, caregiver arrangements, housing, education, employment, co-morbidities, access to reproductive and mental health care, substance use, and pregnancy and pregnancy outcomes.
- Tier three or “research variables” were defined as those unlikely to be routinely collected and would likely need to be determined in the context of targeted research. These include variables capturing detailed mental health (e.g., depression screens), social well-being (e.g., resilience factors), sexual health, and HIV resistance data.

The selected variables were then defined, formatted, and organized into tables. These tables were added to and harmonized with the existing IeDEA DES [20] to create the GRADUATE DES. To facilitate patient-level data collection of variables included in the GRADUATE DES, we designed a folder review form to abstract information from paper-based and electronic routine medical records and databases, as well as self-administered and interview-based questionnaires for completion by the participant or by the study staff while interviewing the participant, respectively.

### Phase 3: Collection of transition-related information using GRADUATE data collection tools

We conducted a cross-sectional study from 1 March to 31 July 2019 of AYLH aged 18–24 years and in care at participating IeDEA sites to 1) test the utility of the GRADUATE DES and associated data collection tools within routine care, 2) evaluate the proportion of key variables in describing the processes, predictors, and outcomes of transition available in routine medical records, 3) examine the proportion of these variables not available that can be obtained from additional data collection efforts, such as through questionnaires, and 4) compare the completeness and quality of transition-related data across participating sites. The study was implemented at sites with a clearly delineated process for transitioning youth to adult HIV care: one primary care clinic (Lighthouse Trust Clinic, Malawi) and three hospitals (Rahima Moosa Mother and Child Hospital, South Africa; Siriraj Hospital and Srinagarind Hospital, Thailand).

- Lighthouse Trust Clinic is a primary care facility in Kamuzu Central Hospital in Lilongwe, Malawi, which manages patients living with HIV from childhood through adolescence and into adulthood. As they transition from paediatric to adolescent to adult care, patients within this facility are seen in separate clinics (e.g., on different days of the week) using established guidelines for transition, making it highly feasible to identify the shift from paediatric to adolescent care in the data.
- The Empilweni Services and Research Unit within the Rahima Moosa Mother and Child Hospital in Johannesburg, South Africa offers routine maternity, neonatal and paediatric services. It is one of the largest paediatric HIV treatment clinics in Johannesburg, South Africa, managing infants and children with HIV or who are HV-exposed. Healthcare transition for AYLH involves them being transferred to a different facility with adult HIV care services. Transition is discussed with the adolescent during up to three visits before the planned transition, so they can discuss with family and decide which facility they would like to transition to. On the day of transition, adolescents are given a transfer letter that contains details of ART initiation, regimens and switches, blood results at initiation and transfer as well as other important clinical or psychosocial information.
- Siriraj Hospital, Mahidol University in Bangkok, Thailand is a pediatric and adult HIV referral center, offering a dedicated sexually transmitted infections and HIV clinic. HIV pediatric to adult healthcare transition involves transfer of care to a different facility based on geographic proximity to the patient’s home using established guidelines for preparing and implementing transition through a comprehensive youth program called the “Happy Teen Program,” which has previously been described [22].
- Srinagarind Hospital in Khon Kaen, Thailand, is a tertiary hospital offering specialist pediatric and adult HIV services. Healthcare transition for AYLH involves transfer of care to a different facility based on geographic proximity to the patient’s home [23].

AYLH within these facilities with the following criteria were eligible for enrolment into the pilot study: aged between ≥18 to ≤24 years of age (at the date of consent); have confirmed HIV infection; fully disclosed to regarding their HIV infection status; with transition either scheduled to take place within the next year, or currently transitioning, or recently transitioned to adult HIV care within the previous one year. At all four facilities, routine electronic and paper-based medical records for enrolled participants were reviewed and anonymized data abstracted into the folder review form. In addition, study staff administered interview-based questionnaires to participating AYLH at Lighthouse Trust Clinic. Participants at Rahima Moosa Mother and Child Hospital completed self-administered questionnaires.

An online Research Electronic Data Capture (REDCap) data management platform was created using the REDCap tools hosted by the University of Cape Town. REDCap is a secure, web-based software platform designed to support data capture for research studies, providing 1) an intuitive interface for validated data capture; 2) audit trails for tracking data manipulation and export procedures; 3) automated export procedures for data downloads to common statistical packages; and 4) procedures for data integration and interoperability with external sources [24,25]. All information obtained was anonymized and entered onto the online data management platform, with each site being able to view and having access only to their data, and the data center having access to only anonymized data from all the sites.

Each site received ethics approval from their local Institutional Review Boards to conduct the pilot study and submit de-identified data for analysis, namely the National Health Sciences Research Committee of the Malawi Ministry of Health and Population, Rahima Moosa Mother and Child Hospital Institutional Review Board, Siriraj Institutional Review Board, and Khon Kaen University Ethics Committee for Human Research. The Centre for Infectious Disease Epidemiology and research (CIDER) at the University of Cape Town received ethics approval from the University of Cape Town Faculty of Health Sciences Human Research Ethics Committee to receive, merge and analyze the de-identified data. Written informed consent was required for all participants from Lighthouse Trust clinic and Rahima Moosa Mother and Child Hospital but was not for Siriraj and Srinagarind Hospitals, as their data used in the analysis were collected during routine care and their relevant IRBs waived consent for this research.

### Statistical analysis

A descriptive analysis was done of the demographic, socio-economic, clinical, and laboratory characteristics of AYLH transitioning to adulthood across the sites and of transition-related processes and outcomes across settings. Key characteristics described and compared include sex, age at transition, duration in HIV care/on ART, likely route/timing of infection, regimen, adherence, disclosure, clinical, immunological and virologic status, education, housing, employment, incarceration, pregnancy, hospital admissions, mental health referrals and diagnosis, and preparedness for and attitude towards transition process. Covariates of interest were described, and bivariate associations explored, according to distributional patterns. The McNemar test was used to determine if there was a significant difference in the proportion of participants with data available when using routine medical records compared to when using data obtained through questionnaires.

All data were analyzed using Stata Version 15.0 (Stata Corporation, College Station, Texas).

### Inclusivity in global research

Additional information regarding the ethical, cultural, and scientific considerations specific to inclusivity in global research is included in the Supporting Information (S1 Checklist).

## Results

We identified 114 variables as essential to characterizing AYLH transitioning to adulthood within LMICs and capturing transition-related processes and outcomes. These include variables capturing socio-demographic characteristics, medication responsibilities, disclosure, adherence, substance and alcohol use, smoking, sexual history, laboratory findings, fractures, hospitalization, self-harm, mental health diagnosis, referral or treatment, transfers, and transition experiences (Table 1).

**Table 1:** Variables identified as key in capturing the characteristics of adolescents and young adults living with HIV transitioning to adulthood across LMICs, as well as the transition-related processes and outcomes.

Theme	Variable
Socio-demographic	• Date of birth • Sex • Living arrangements • Type of dwelling currently live in • Longest duration of homelessness if any • Schooling including currently attending any formal school, attending secondary school • Current employment situation including any full-time or part-time work • Main source of income including any receipt of social grants • Episodes of incarceration (>1 day in prison) as an adolescent or young adult (ages 10–25 years), including age, number of separate incidents, and duration. • Vital status of biological mother and father • Current relationship status • Date of birth, HIV status, and HIV disclosure status of any biological children
Disclosure	• Disclosure status, process and age, including self-disclosure to others
ART medication clinic visits	• Date of patient’s visit • Type of clinic in terms of age group where patient is seen • Person responsible for making travel arrangements to get to clinic appointments and medicine collection. • Person responsible for patient taking ART medications.
ART adherence	• Time period over which ART adherence variables are asked. • Method of adherence measurement used and adherence (perfect vs imperfect)
Medical history	• Laboratory measures • Hospital admissions including date of admission and discharge, diagnosis, and any intensive care admissions • Mental health referrals, diagnoses, and treatment • History of fracture(s) including site and level of trauma that caused the fracture
Recorded outcome	• Dropping out of care and reasons • Patients’ vital status
Alcohol intake	• Alcohol intake including age at which they first drank a whole unit of alcohol, frequency of drinking, and amount of alcohol units consumed on a typical day, binge drinking.
Smoking	• History of smoking, including age at which they first smoked a whole cigarette and frequency of smoking
Recreational drugs	• Use of non-injectable and injectable recreational drugs other than cigarettes and alcohol, including age at which they first started using and frequency of use
Sexual activity (only for children >12 years of age)	• Primary sexual preference • Sexual activity history including number of sexual partners, type of encounter (vagina, oral, anal), and frequency of condom use,
Self-harm	• Episodes of self-harm in the past 12 months including method(s) used
Transfer	• Date of transfer out • Did the patient attend a visit at the facility to which they were transferred? • Date of transfer in • Was this transfer referred by the transfer out facility or was it a self-referral/silent transfer that the transfer out facility was not aware of or did not initiate? • Primary (main) reason for transfer • Other reason for transfer (Additional reasons for transfer e.g., patient moves to another area and commences antenatal care) • Program from which patient was transferred out
**Transition experience (paediatric to adolescent or adolescent to adult transition, with or without a shared care clinic)**	
Pre-transition	• How many weeks before last date at the transferring out clinic was the patient first told about transfer? • Number of clinic visits at which transfer was discussed prior to visit at which patient was transferred out? • Age of patient in years when first discussed transition to another clinic. • Did patient have a written transition/transfer plan before transfer (e.g., brochure, checklist, webpage, adolescent “passport”) • Who brought the patient to the clinic visit immediately after transfer (i.e., the transfer in visit)?
Post-transition (6–12 months after the first visit to the new clinic/facility)	• Date on which post-transfer/transition questions are asked? • How prepared/unprepared did the patient feel for transfer/transition • How easy/difficult was the transfer/transition? • How satisfied/comfortable is patient with post-transfer clinic? • How satisfied/comfortable is patient with staff at post-transfer clinic

Of the 100 AYLH included in the pilot-study (52% female), 60 (53% female) were from the African sites (30 from Lighthouse Trust clinic and 30 from Rahima Moosa Mother and Child Hospital) and 40 (50% female) were from sites in Asia (20 from Siriraj Hospital and 20 from Srinagarind Hospital). The median age at enrolment (interquartile range [IQR]) was 20.7 (19.2; 21.8) years. The youngest participant was aged 15 years and the eldest was 27 years (Table 2). All the participants with data available reported having been perinatally exposed to HIV and all were currently on ART. The median (IQR) age at which the participants became fully aware of their HIV status was 12 (11; 14) years with half (50.5%; 95% CI 40–61%) having been informed of their HIV status by a healthcare worker, 41% (95% CI 31–52%) by their caregiver and 6.2% (95% CI 2.3–13%) having worked it out themselves before being informed. One-third had been referred for a mental health diagnosis or treatment (32%; 95% CI 22–43%) or had been hospitalized (38%; 95% CI 28–48%) during adolescence (Table 2).

**Table 2:** Description of 100 adolescents and young adults living with HIV included in the pilot study to evaluate the proportion of variables identified as key in describing the processes, predictors, and outcomes of transition available in routine medical records and examine the ease of obtaining these variables through questionnaires.

Characteristic	African sites^#^		Asian sites^##^		Total	
Number	60		40		100	
Age (years), median (IQR)	20.2	(18.8; 21.9)	21.1	(19.8; 21.5)	20.7	(19.2; 21.8)
Females, n (%)	32	(53)	20	(50)	52	(52)
Single or double orphaned, n/N (%)	50/60	(83)	27/38	(71)	77/98	(79)
Currently attending any formal schooling, n/N (%)	47/60	(78)	20/38	(53)	67/98	(68)
Currently employed, n/N (%)	9/60	(15)	12/21	(57)	21/81	(26)
Dependent on self as main source of income Self, n/N (%)	11/60	(18)	6/18	(33)	17/78	(22)
Has a biological child, n/N (%)	3/60	(5)	2/38	(5)	5/98	(5)
Perinatal HIV exposure (self-reported), n/N (%)	52/52	(100)	38/38	(100)	90/90	(100)
Age (years) at which became fully aware of HIV status, median (IQR)	13.0	(11.0; 15.0)	12.0	(11.0; 13.0)	12.0	(11.0; 14.0)
Told by caregiver, n/N (%)	36/60	(60)	4/37	(11)	40/97	(41)
Told by health care worker, n/N (%)	16/60	(27)	33/37	(89)	49/97	(51)
Worked out HIV status, n/N (%)	6/60	(10)	0		6/97	(6)
Has disclosed HIV status to others excluding family members, n/N (%)	29/59	(49)	15/20	(75)	44/79	(56)
Currently on ART, n/N (%)	60/60	(100)	39/40	(98)	99/100	(99)
Attended last clinic visit without any caregiver, n/N (%)	50/60	(83)	6/20	(30)	56/80	(70)
Responsible for making own travel arrangements to the clinic always, n/N (%)	35/60	(58)	19/20	(95)	54/80	(68)
Responsible for taking own ART medications always, n/N (%)	51/60	(85)	23/25	(92)	74/85	(87)
Ever been referred for a mental health diagnosis or treatment since the age of 10 years, n/N (%)	17/60	(28)	8/19	(42)	25/79	(32)
Episode of self-harm in the past 12 months, n (%)	3/59	(5.0)	0/40?		3/67	(5)
Ever been hospitalized since the age of 10 years, n/N (%)	28/60	(47)	9/38	(24)	37/98	(38)
Ever had a bone fracture since the age of 10 years, n/N (%)	16/59	(27)	1/21	(5)	17/80	(21)
Ever smoked a whole cigarette, n/N (%)	13/59	(22)	6/16	(38)	19/75	(25)
Ever drunk a whole unit of alcohol, n/N (%)	35/60	(58)	6/15	(40)	41/75	(55)
Ever used injectable or non-injectable recreational drugs, n/N (%)	2/60	(3)	0/40		2/100	(2)
Ever engaged in vaginal, anal, or oral sex, n/N (%)	38/60	(63)	11/21	(52)	49/81	(61)
Transitioned to adult care*, n (%)	32	(53)	39	(98)	71	(71)
Age at transition (years), median (IQR)	20.6	(20.2; 21.9)	19.7	(18.9; 20.5)	20.2	(19.4; 21.2)
Transferred care as part of transition, n/N (%)	2/32	(6)	39/39	(100)	41/71	(58)
Pre-transition discussion prior to transition, n/N (%)	32/32	(100)	35/39	(90)	67/71	(94)
Felt somewhat prepared or very prepared for the transition/transfer, n/N (%)	26/32	(81)	18/20	(90)	44/52	(85)
Feel the transition/transfer was somewhat easy or very easy, n/N (%)	20/32	(63)	N/A		20/32	(63)
Very or somewhat satisfied/comfortable with the post-transfer clinic, n/N (%)	20/29	(69)	N/A		20/29	(69)
Very or somewhat satisfied/comfortable with the staff at the post-transfer clinic, n/N (%)	20/29	(69)	N/A		20/29	(69)

^#^ Participants from Lighthouse Trust Clinic in Malawi (n = 30) and Rahima Moosa Mother and Child Hospital in South Africa (n = 30). Data obtained from routine medical records as well as from additional data collection through questionnaires.

^##^ Participants from Siriraj Hospital (n = 20) and Srinagarind Hospital (n = 20) in Thailand. Data obtained from routine medical records only.

* Definition of transition varies across the sites.

In total, 71% (53% in African sites vs. 97.5% in Asian sites) had transitioned/transferred to adult care, as per the definition of each site. Of these, 30 (42%) of the participants transitioned from an adolescent-specific model of care within an integrated family clinic to having their clinic visits scheduled on a different day of the week while 41 (58%) transferred from a pediatric facility to a different facility offering adult HIV care. Median (IQR) age of transition/transfer was 20.6 (20.2; 21.9) years in African sites and 19.7 (18.9; 20.5) years in the Asian sites (Table 2). Ninety-four percent (95% CI 86–98%) of those who had transitioned/transferred to adult care had a transition-related discussion with their healthcare providers prior to the transition, 85% (95% CI 72%-93%) felt somewhat prepared or very prepared, 62.5% (95% CI 44–79%) felt that the transition/transfer was somewhat easy or very easy, and 69% (95% CI 49–85%) were somewhat or very satisfied/comfortable with the post-transfer clinic and the staff (Table 2).

**Fig 2:**
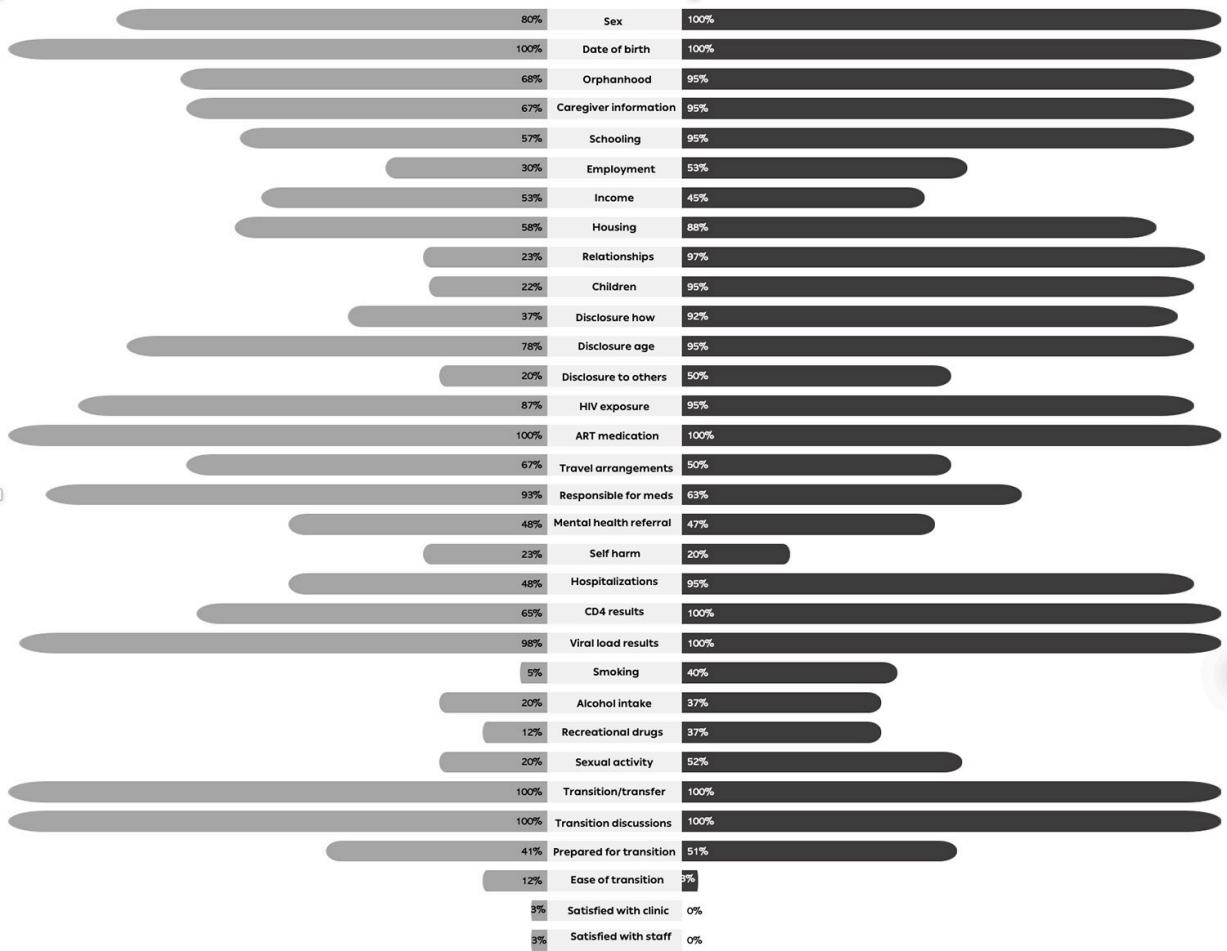
Proportion of participants with data available within their routine medical records across the various themes.

Information on sociodemographic variables (sex and date of birth), ART medication history, viral load results, HIV exposure, and whether the adolescent had transitioned/transferred care and had prior discussions were available in ≥80% of routine medical records across both African and Asian sites (Fig 2). Information that varied greatly in availability across the sites, and with significantly less recording of this information within routine medical records for participants in the African sites, included that relating to recording of relationship status (African vs. Asian sites; 23% vs. 97%; difference -74%, p <0.01), biological children (22% vs. 95%; difference -73%; p <0.01), who had disclosed their HIV status to them (37% vs. 92%; difference -55%; p <0.01), history of hospitalizations (48% vs. 95%; difference -47%, p <0.01), smoking (5% vs. 40%), sexual activity (20% vs. 52%; difference; p <0.01).

For both African and Asian sites, there was incomplete recording in routine medical records of information relating to satisfaction with post-transition clinic (3% and 0%) and staff (3% and 0%), ease of transition (12% and 3%), self-harm (23% and 20%), smoking (5% and 40%), use of recreational drugs (12% and 37%), alcohol intake (20% and 37%), referral for mental health diagnosis or treatment (48% and 47%), sexual activity (20% and 52%), and level of preparedness for transition (41% and 51%), respectively (Fig 2).

Additional data collection through interviewer-based and self-conducted questionnaires was only conducted in the African sites. All participants within these sites completed the questionnaires. There was a significant increase across all variables in the data availability upon eliciting information through questionnaires (Table 3), with 100% availability noted across all variables included in the questionnaires. Most significant improvements could be noted for most transition-related variables such as feeling prepared for transition, ease of transition, and satisfaction with post-transition clinic and staff.

**Table 3:** Proportion of participants with data available within routine medical records compared to data that became available upon collecting additional data through questionnaire (N = 60) among participants in African sites.

Characteristic	Proportion with data available*, n/N (%)		Difference %		(95% CI) **	p-value
Sex	48/60	(80)	20%	(9.9–30)		<0.01
Orphanhood	41/60	(68%)	32%	(20–44)		<0.01
Caregiver information	40/60	(67%)	33%	(21–45%)		<0.01
Currently attending any schooling	34/60	(57%)	43%	(31–56%)		<0.01
Currently employed	18/60	(30%)	70%	(58–82%)		<0.01
Sources of income	32/60	(53%)	47%	(34–59%)		<0.01
Housing	35/60	(58%)	42%	(29–54%)		<0.01
Current relationship status	14/60	(23%)	77%	(66–87%)		<0.01
Biological children	13/60	(22%)	78%	(68–89%)		<0.01
How they became fully aware of HIV status (disclosure how)	22/60	(37%)	63%	(51–75.5%)		<0.01
Disclosure age	47/60	(78%)	22%	(11–32%)		<0.01
Disclosure of HIV status to others excluding family members	12/60	(20%)	80%	(70–90%)		<0.01
HIV exposure	52/60	(87%)	13%	(4.7–22%)		<0.01
Responsible for making own travel arrangements to the clinic always	40/60	(67%)	33%	(21–45%)		<0.01
Responsible for taking own ART medications always,	56/60	(93%)	6.7%	(0.4–13%)		0.04
Ever been referred for a mental health diagnoses or treatment during adolescence	29/60	(48%)	52%	(39–64%)		<0.01
Ever hurt themselves on purpose in the last 12 months (self-harm)	14/60	(23%)	77%	(66–87%)		<0.01
Hospitalizations since the age of 10 years	29/60	(48%)	52%	(39–64%)		<0.01
Smoking	3/60	(5.0%)	95%	(89.5–100%)		<0.01
Alcohol	12/60	(20%)	80%	(70–90%)		<0.01
Use of recreational drugs	7/60	(12%)	88%	(80–96%)		<0.01
Sexual activity	12/60	(20%)	80%	(70–90%)		<0.01
Feeling prepared for transition/transfer	13/32	(41%)	59%	(42–76%)		<0.01
Ease of transition/transfer	4/32	(12.5%)	87.5%	(76–99%)		<0.01
Satisfaction with post-transition clinic	1/32	(3.1%)	97%	(91–100%)		<0.01
Satisfaction with post-transition clinic staff	1/32	(3.1%)	97%	(91–100%)		<0.01

* Data available only within routine medical records.

** Difference when comparing data available within routine medical records to that availability (100% across all variables) with additional data collection through questionnaires.

## Discussion

We showed that it was feasible for a team of technical experts to agree on a set of variables and definitions that are key to measuring transition and transition-related outcome—despite managing AYLH across diverse geographic, economic, and social landscapes, and with varying definitions of adolescent to adult HIV care transition. Leveraging work already conducted by IeDEA, we were able to integrate the identified variables into the IeDEA DES to create a comprehensive set of data tables that can be used to extract information needed to describe and characterize AYLH transitioning to adult care, and to study transition-related processes and outcomes across LMICs more fully.

The GRADUATE project at least partly addressed the urgent need for the development and validation of robust and consistent measures of transition readiness, processes, and outcomes to allow interstudy comparison as well as to consistently report outcomes across varying settings [26–31]. Using the additional data collection through the GRADUATE harmonized set of data tools, we were able to characterize the health and associated social determinants of AYLH transitioning to adulthood across our sites, and document key aspects of transition preparation, implementation, and outcomes more fully. We were able to gain insights into key domains known to be associated with transition readiness–disclosure, health navigation, self‐advocacy, and health literacy [32–34]. While the subject of healthcare transition is not new and has been noted for adolescents with other chronic diseases, there are still very few studies that examine transition measures and associated health conditions, which may be critical to achieving optimal disease specific outcomes. For example, there was very poor recording of information relating to referral for mental health diagnosis or treatment, self-harm, smoking, alcohol intake, use of recreational drugs, and sexual activity in routine health records, despite the fact that AYLH are known to be at risk of developing mental health complications, including depression, anxiety, substance use and post‐traumatic stress disorder (PTSD), which may all lead to self-reported non-adherence and missed ART doses as well as unsuppressed VL and poor treatment outcomes [35–40]. Regular assessment, documentation and management of any conditions that may have a negative impact on adherence to medications is critical in AYLH, especially as they transition to adulthood when AYLH are known to have sub-optimal adherence [41]

The World Health Organization (WHO) emphasizes the importance of integrating HIV care with other relevant services, such as sexual and reproductive health, which has been found to be feasible across a variety of settings and integration models [42–44]. This integration is also important to providing youth-friendly, "one-stop" health care delivery services to facilitate efficient uptake of key services [45]. Therefore, the very poor recording of sexual health information observed in our study (e.g., sexual activity, consistency of condom use), suggests that true integration of services is limited. Also of concern was the poor recording of any biological children of ALYH among sites in Africa, with only one-fifth of participants having a record of this being documented at their HIV clinics.

In our study we included questions on when the process of disclosure started, age when HIV status was fully disclosed, who disclosed their HIV status, and non-family members to whom they had disclosed their status to. While age of disclosure was well documented within routine medical records, less than half of participants had information on disclosure practices documented in their routine medical records. Given that disclosure is known to be an important component of ART adherence and retention in care [46–49], as well as being a key prerequisite to transition, it is important for the disclosure process to be better documented by HIV care providers.

Inclusion of transition-related variables within local administrative and national HIV program databases would be valuable for conducting transition assessments, as it captures data from individuals who might otherwise not respond to surveys or participate in research, makes data easily accessible with limited additional costs, and facilitates linkage with other databases [30]. Despite consistent recording of the pre-transition discussions, less than half of records across all the sites included any information on the transition and post-transition experience (e.g., individual perceptions of preparedness for the process, satisfaction with new clinics and providers). In our interviews, participants were forthcoming with this information once they were asked to do so.

Also of concern is that the interview responses showed that even though >80% felt prepared or somewhat prepared for transition, only two-thirds described their transition as easy or being satisfied with the post-transfer clinic and staff. Following up with ALYH after transition is still part of the overall process, and soliciting their feedback could be valuable to informing future improvements [26]. Given that this is a critical phase of their lives, discussions around how AYLH are feeling are important to encourage them and understand barriers they are facing to continued care, and to guide the scope and depth of pre-transition discussions to make the process easier. Knowing whether AYLH are satisfied and comfortable with their new clinic and providers could assist with developing retention strategies to avoid clinic drop-out and improve outcomes, but very little data exist within routine medical records to fully describe this.

Central to assessing and comparing transition outcomes is knowing how to optimally measure this process and implementing data collection across different settings. There is growing recognition of the need for data harmonization strategies. However, global programs currently set up to harness and improve large datasets have not focused on variables to capture transition preparation and implementation, and post-transition outcomes. Also, there are no mechanisms within current global surveillance data systems to distinguish what proportion of young people living with HIV have transitioned from paediatric to adult HIV care and when this is occurring [50]. Unless the often-fragile healthcare systems delivering HIV care adapt to capture these outcomes, their data will be lost. If this population cannot be counted and reported, it may become less urgent for policy makers to support interventions to improve adolescent HIV outcomes. A model for gathering transition-related data in LMICs would also further global efforts to track adolescents moving out of paediatric care and promote age disaggregation in HIV surveillance [51].

Our study is especially relevant as it was conducted in public sector ART clinics in South Africa, Malawi, and Thailand, and most adolescents living with HIV reside in sub‐Saharan Africa and receive care in similar healthcare settings. Furthermore, participants enrolled in our study received only routine services that were reflective of the local standard of care for adolescent ART patients. Evaluating availability of information both within the paper-based medical records as well as electronic-records is another study strength. Nonetheless, our results should be interpreted in light of several limitations. Generalizability of our findings is limited as the pilot was conducted at only four sites, using a cross-sectional design with convenience sampling among mostly stable patients on ART. We were not able to conduct interviews in the Asian sites, and so our characterization and assessment of the utility of the questionnaires for this population were incomplete. The data obtained from interviews were based on self-report and may be subject to recall and desirability bias. Accuracy of self-reported medical history data was not confirmed with medical records.

## Conclusion

We developed measures of transition readiness, processes, and outcomes that can be valuable resources across varying geographic areas and care delivery models. There is a need to improve the recording of transition-related variables within routine records and national HIV care databases to optimally measure transition preparation, implementation, and outcomes.

## Supporting information

S1 Checklist Ethical, cultural, and scientific considerations specific to inclusivity in global research. (DOCX)
